# Improving perioperative management of surgical sets for trauma surgeries: the 4S approach

**DOI:** 10.1186/s12913-022-08671-2

**Published:** 2022-10-28

**Authors:** Julio Ribes-Iborra, Borja Segarra, Victor Cortés-Tronch, Javier Quintana, Thibaut Galvain, Christian Muehlendyck, Elena Escalona, Suzanne Battaglia, Jorge Navarrete-Dualde

**Affiliations:** 1 Orthopedic Department, Hospital Universitario de La Ribera, Alzira, Valencia Spain; 2 Johnson & Johnson MedTech, New Brunswick, NJ USA

**Keywords:** Surgical set, Surgical tray, Instruments, Implants, Perioperative management, Standardization, Sterility, Safety, Stock management, 4S program

## Abstract

**Background:**

The perioperative management of the surgical instruments and implants that comprise sets for trauma surgeries has been identified as a complex and resource-intensive activity due to non-standardized inventories, redundant surgical instruments and unnecessary sterilization cycles. The 4S Intelligent Trauma Care program aims to improve perioperative management and thereby reduce environmental impact by utilizing standardized inventories, a sterile implant portfolio, a barcode that enables a digital safety certification, and a digitized restocking service.

**Objective:**

The objective of this study was to investigate the impact of the introduction of the 4S program for the management of surgical sets in open reduction internal fixation (ORIF) trauma surgeries.

**Methods:**

This was a single-center, quality improvement study of ORIF trauma surgeries, comparing the current practice (30 procedures) to the procedure following the introduction of the 4S program (30 procedures). The primary outcome was the proportion of procedures requiring only one sterilization cycle. Secondary outcomes were the number of sterilization cycles per procedure, set processing time across departments, total set processing costs, number of missing or damaged implants, number of cleaning cycles per procedure, time taken to assemble containers for sterilization, number of containers entering the autoclave per procedure, environmental impact, number of baskets entering the cleaning machine per procedure, and staff satisfaction.

**Results:**

Introduction of the 4S program resulted in a reduction in the mean number of sterilization cycles required from 2.1 to 1.0 (p < 0.001). In the current practice, only 30.0% of procedure sets were sterilized within one cycle, compared to 100.0% following introduction of the 4S program (p < 0.001). A reduction in the mean set processing time of 24.1% in the OR and 35.3% in the sterilization department was observed. Mean set processing costs for the current practice were €81.23, compared to €50.30 following introduction of the 4S program. Furthermore, following the introduction of the 4S program, procedures were associated with significant reductions in water and electricity usage, and increased staff satisfaction.

**Conclusions:**

This quality improvement study demonstrates the substantial time and cost savings, positive environmental impact and staff satisfaction that could be achieved by streamlining surgical set management through the 4S program. To our knowledge, this is the first study of this type and our findings may be instructive to other hospitals and surgical specialties.

## Introduction

The perioperative management of surgical instruments and implants has been identified as a complex and resource-intensive activity frequently associated with substantial administrative and financial burden. [1–4] In our experience, ahead of a traumatological surgical procedure, instruments and implants to be used in the operating room (OR) are placed inside trays which are then deposited in metal containers to create surgical sets. After surgery, all instruments and unused implants require reprocessing, which involves disassembly of instruments, cleaning, disinfection, inspection and functional testing, repackaging and sterilization. Contaminated instruments and clean but unused implants have to be cleaned separately from each other to avoid any potential contamination of implants. Following this, further sterilization cycles may be needed to add required implants to the trays.

Underutilization of instruments during surgery has been observed in multiple studies, likely resulting in unnecessary reprocessing of unused equipment. [1, 3–7] In one study investigating otolaryngology, plastic surgery, bariatric surgery and neurosurgery, the average instrument use per tray varied from 13–22%, demonstrating that the majority of included instruments were most often unnecessary. [4].

In traumatology, the inclusion of implants alongside instruments within surgical sets introduces additional complexity. In our experience, frequently, implants are added after the initial sterilization of instruments as they were not on site at the time of the first sterilization, resulting in the requirement for an additional sterilization cycle. EU Medical Device Regulations (MDR) enforce traceability requirements for all implants used. [8] Commonly used paper-based methods of registering and recording implant use can result in a substantial resource burden and the potential for human error. Furthermore, in our experience with the current practice, it is not possible to track which implants are used in a given procedure as they are added into surgical sets ahead of use, as well as potentially undergoing multiple rounds of reprocessing.

The absence of standardized inventories, the redundancy of and in surgical sets and unnecessary sterilization cycles all contribute to an increase in health resource utilization, and raise concerns over environmental impact and sustainability of these practices. [1–5, 7] This is anticipated to be particularly acute in trauma surgery due to the wide variation of specialized implants potentially required and because surgical sets for trauma surgeries traditionally include plates and screws alongside instruments. There is a clear need for a system that provides organized and immediately available instruments and implants for use in time-critical and often unscheduled procedures.

Previous cost and quality improvement studies have shown that the introduction of programs to improve the management of surgical sets have resulted in substantial cost and time savings via reductions in instrument reprocessing. [5, 6, 9, 10] However, there are no known quality improvement studies that assess the impact of the introduction of a program designed to improve surgical set management for trauma surgeries across all departments (OR, sterilization and purchasing departments). To this end, the 4S Intelligent Trauma Care (Johnson & Johnson MedTech, New Brunswick, New Jersey, USA; referred to herein as 4S) program has been devised to include the following four components:

1. **Standardized Inventory**: Surgical sets are standardized to create new lightweight versions that contain the instruments only, eliminating all implants (plates and screws). These smaller sets are designed to be used across a wide range of traumatological surgical indications, creating a simplified inventory and removing the need for procedure-specific sets.
2. **Sterile Portfolio**: Pre-sterilized, individually packed, ready-to-use, bar- and color-coded implants separate to the instrument sets are used.
3. **Safety Certification**: Implants can be traced from manufacturers to patients through the use of barcoding and a digital management system, resulting in clear and precise documentation
4. **Service and Advanced Planning**: The program introduces digital management of restocking, reducing personnel time required.

The objective of this study was to investigate the impact of the introduction of the 4S program for the management of surgical sets in open reduction internal fixation (ORIF) surgeries. The first hypothesis was that the introduction of the 4S program would reduce the number of sterilization cycles of the required surgical sets, thereby reducing the environmental impact and increasing the sustainability of surgical set processing. It was further hypothesized that the 4S program would improve perioperative processes by reducing staff and set turnover time, reducing hospital costs, and improving staff satisfaction for activities related to the management of surgical instruments and implants used in ORIF procedures. The reporting of this study was designed to align with the Standards for Quality Improvement Reporting Excellence (SQUIRE) 2.0 guidelines, which provides guidance on reporting systematic efforts to improve the quality, value and safety of healthcare. [11].

## Methods

### Study design

This was a single-center, pre-post quality improvement study of trauma surgeries, comparing procedures performed with the current practice to those performed following the introduction of the 4S program, from November 2019 to November 2020. Both cohorts consisted of 30 trauma procedures and were selected using convenience sampling.

The study was conducted at a tertiary hospital in Spain, serving a population of around 250,000 people. In 2018, approximately 17,000 surgeries were performed, 2,000 of which were trauma surgeries.

Due to COVID-19 restrictions on external personnel access within the hospital, the study was paused for four months between March 2020 and July 2020.

### Procedure data

Data specific to procedures for all consecutive adult patients (aged 18 and older) undergoing ORIF surgeries with locking plates using the DePuy Synthes (a Johnson and Johnson company) Small Fragment System were included. No patient data were collected. There were no exclusion criteria. No pediatrics were included as these patients undergo conservative treatment rather than ORIF surgery.

### Intervention

The intervention was the introduction of the 4S program. The new standardized surgical set was provided by DePuy Synthes (a Johnson and Johnson company). The composition of the implant trays was designed by a dedicated 4S team at the manufacturer, based on four years of consumption data both at the national and customer level in Spain [unpublished data]. The introduction of the 4S program was facilitated by this 4S team in collaboration with hospital personnel. In order to introduce the 4S program, certain resources were required, including multi-purpose instruments, sterile implants, and storage equipment such as cabinets and trolleys. The staff that worked in the department prior to the introduction of the 4S program were identical to those after the intervention was introduced.

**Fig. 1:**
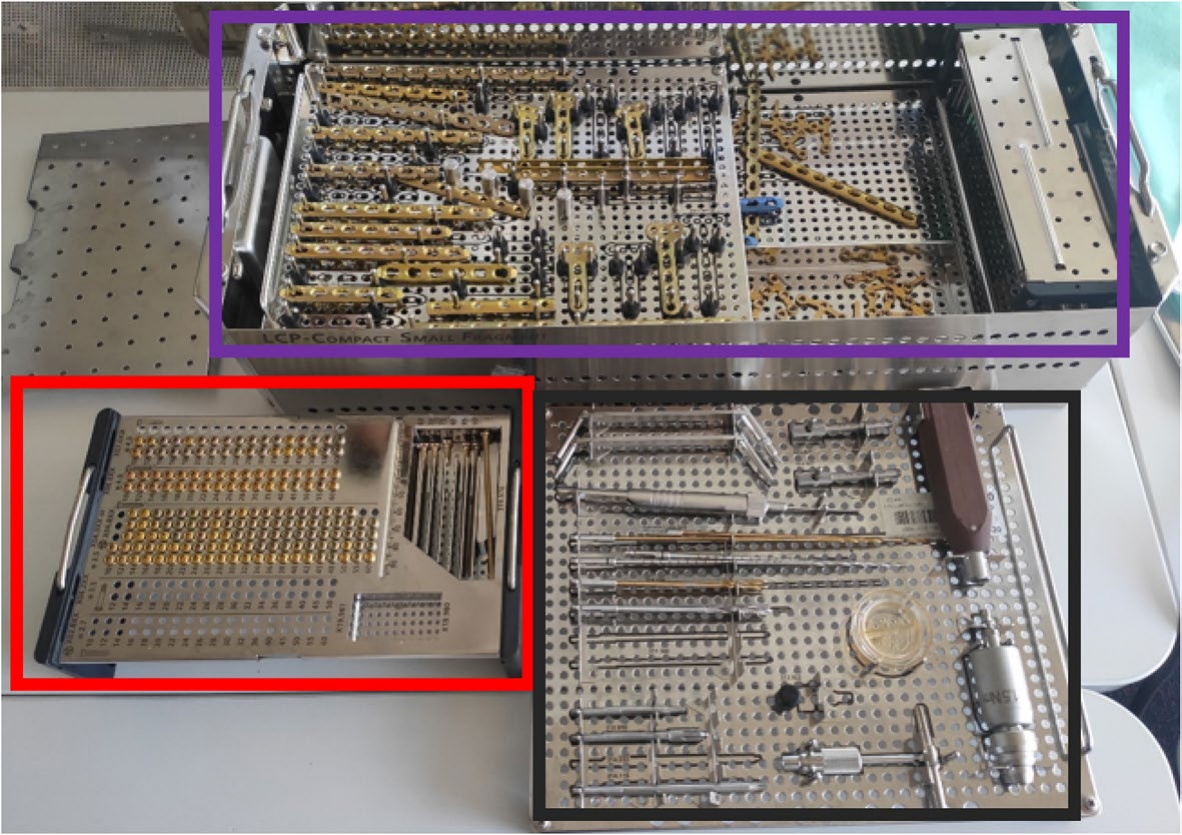
Current practice: Small Fragments set. This encompasses a basic set for small fragment procedures in locations such as the ankle, with no anatomical plates included. Key: purple box: basic plates; red box: screws; black box: instruments (38 in total)

**Fig. 2:**
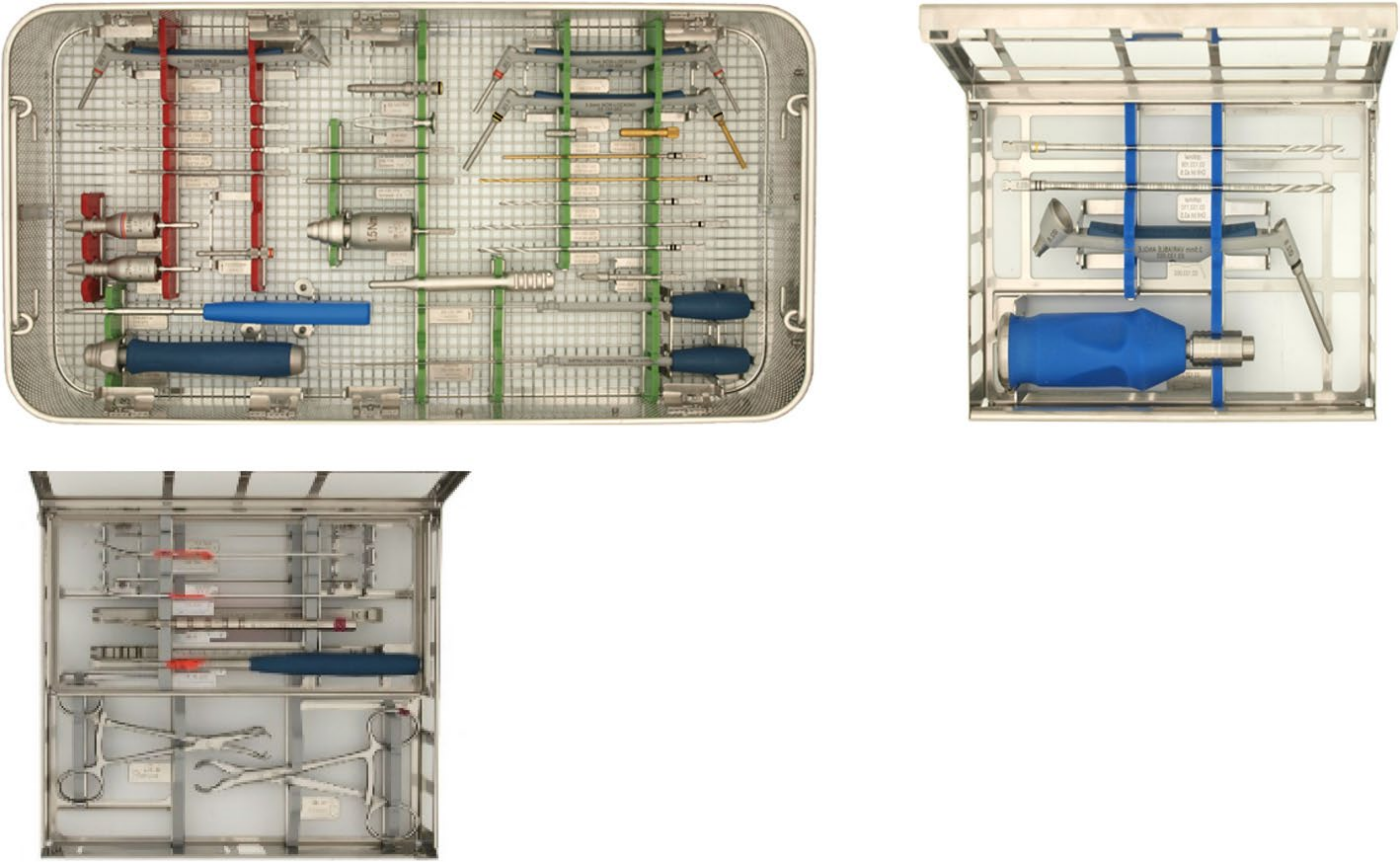
Following introduction of the 4S program: Small Fragments set. This encompasses a set with all instruments related to small fragment surgeries (20 instruments), including those required for anatomical plates such as basic small fragment plates, anatomical ankle or fibula, elbow, clavicle, and distal tibia plates

**Fig. 3:**
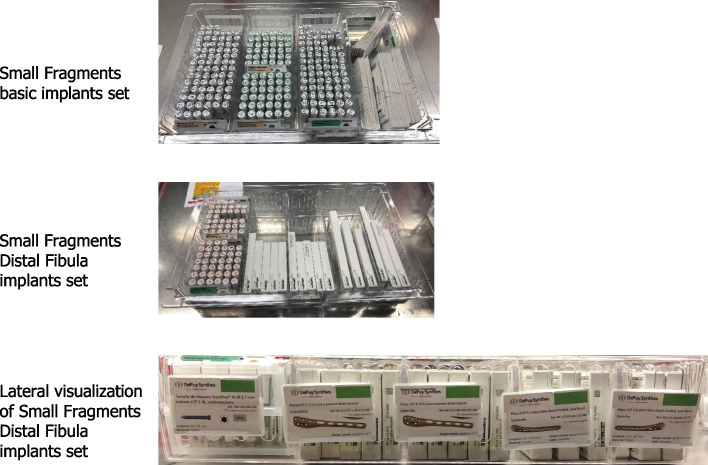
Following introduction of the 4S program: Small Fragments implants (shown in cabinet drawers). Separate pre-sterilized implants for use in conjunction with the Small Fragment set in Fig. 2

**Fig. 4:**
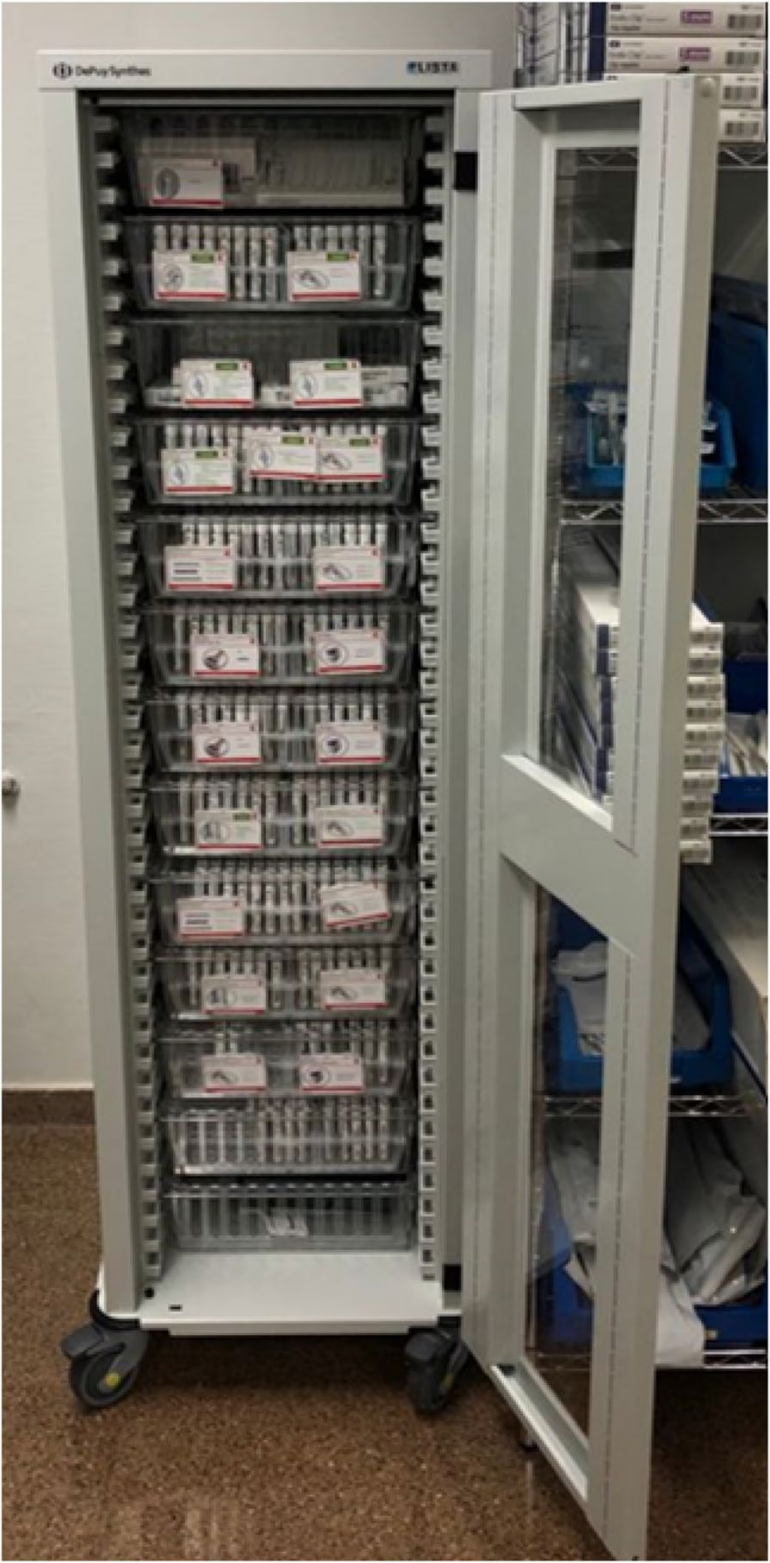
Following introduction of the 4S program: cabinet containing implants. This cabinet includes Small Fragments implants sets together with two distal radius implant sets

Table 1 details the changes made for each of the four components. Photographs of the surgical sets in the current practice, and following the introduction of the 4S program are shown in Figs. 1, 2, 3 and 4.

**Table 1:** Summary of the changes to the current practice following introduction of the 4S program

Management domain	Current practice	Title and details of the 4S program component
Inventory management	Surgical sets included plates and screws Six different surgical sets were utilized in ORIF surgeries (Small Fragments 1 and 2, Elbow, Ankle, PHILOS™ and Tibia [DePuy Synthes, a Johnson and Johnson company])	**Standardized Inventory:** One standardized surgical set (DePuy Synthes, a Johnson and Johnson company) to be used in ORIF surgeries was established with the number of instruments reduced to create a streamlined set All implants (screws and plates) were removed from sets
Implant management	A non-sterile surgical set model was utilized in which all implants were non-sterile upon delivery to the hospital and sterilized prior to surgery Unused implants would undergo reprocessing and re-sterilization	**Sterile Portfolio:** All implants were provided in an individual pre-packaged and sterilized format
Safety management	Traceability of individual implants relied on manual processing at the hospital	**Safety Certification:** All implants were sterilized by the manufacturer and labeled to allow traceability
Stock management	Stock management involved manual processing of data	**Service and Advanced Planning:** Digital management of stock control was done by in situ barcode reading and the use of an advanced inventory management system to digitalize processes (eSIMS Advanced Inventory Management Solution)

### Study endpoints

Data concerning processing times were collected following each activity through in-person direct observation by a trained investigator using a calibrated stopwatch. The investigator was trained on the definitions of each of the outcome measures. The investigator could not be blinded to the intervention in this study, due to the nature of data collection in a pre- and post- study design, which involved a change of practice.

The primary outcome was the proportion of procedures requiring only one sterilization cycle. The secondary outcomes (Table 2) were i) the number of sterilization cycles per procedure; ii) the set processing time in the OR; ii) the set processing time in the sterilization department (including the time taken to assemble containers for sterilization); iii) the set processing time in the purchasing department; iv) the overall set processing time; v) the number of missing or damaged implants per procedure; vi) the number of baskets used per procedure; vii) the number of cleaning cycles required per procedure; viii) the number of containers entering the autoclave; ix) the total set processing costs; x) water consumption and cost; xi) electricity consumption and cost, and xii) staff satisfaction.

**Table 2:** Secondary outcomes

Component	Definition
Number of sterilization cycles per procedure	• Mean number or sterilization cycles required per procedure
Set processing time in the OR	Consists of the following: • Time taken to prepare (opening the set, preparing operating table and checking implants) • Time taken to clean and remove systems in the OR (from removal of first implant from the OR to removal of all contaminated sets) • Time taken to record implants used per procedure • Time taken to replenish implants missing in the set
Set processing time in the sterilization department	Consists of the following: • Time taken to wash all instruments and implants per set per procedure • Time taken to assemble cleaning baskets per set per procedure • Time taken for quality inspections before and after mechanical cleaning and assembly of the containers for sterilization per set per procedure
Set processing time in the purchasing department	Consists of the following: • Time taken for validation and coding of the implants used per procedure • Time taken for registration and re-coding time of the implants used per procedure • Time taken to place an implant order per procedure • Time taken to receive and reconciliate implants • Time taken for manual error correction per procedure
Overall set processing time	• Sum of set processing time in the OR, sterilization department and purchasing department
Number of missing or damaged implants per procedure	• Total number or implants unavailable or damaged per procedure
Number of baskets used per procedure	• Number of baskets placed into the cleaning machine per procedure per cycle
Number of cleaning cycles per procedure	• Mean number of cleaning cycles required per procedure
Number of containers entering autoclave	• Mean number of containers entering the autoclave per procedure per cycle
Total set processing costs	Calculations based on the following: • Each cycle (washing, sterilization and consumables) costs €18 (USD$19) • Time costs calculated by costs of relevant workforce per time multiplied by average processing times • Total cost calculated by the sum of the cost of cycles, cost of washing and sterilization time, cost of OR time and cost of purchasing department time
Water consumption and cost	Calculations based on the following: • A water consumption of 60 L per cycle for the cleaning machine and 240 L per cycle for the autoclave • Water consumption calculated as number of cleaning machine cycles multiplied by 60, plus the number of autoclave cycles multiplied by 240 • Costs were based on an average price of water for domestic use in Spain of 1.90 €/m3 (USD$2), as reported in the Asociación Española de Abastecimientos de Agua y Saneamiento—Asociación Española de Empresas Gestoras de los Servicios de Agua Urbana (AEAS-AGA) 2020 tariff survey(17)
Electricity consumption and cost	Calculations based on the following: • An electricity consumption of 0.41 kW per cycle for the cleaning machine and 5 kW per cycle for the autoclave • Electricity consumption calculated as number of cleaning machine cycles multiplied by 0.41, plus the number of autoclave cycles multiplied by 5 • Costs were based on an average price of €0.1214 (USD$0.1279) per kWh, as reported by FACUA in 2020(18)
Staff satisfaction	• Improvement by changing process assessed using a questionnaire • NASA-TLX used to assess staff with physically demanding work

Staff satisfaction was evaluated through two methods. In order to assess improvement by changing processes, a questionnaire developed in The Netherlands was used. [12] For staff with physically demanding work, the NASA Task Load Index (NASA-TLX) was used. [13] NASA-TLX is a widely used mental, multidimensional tool that enables workload to be sensitively and reliably estimated. [13] The assessment method captures the subjective experience of workers engaged in human–machine complex socio-technical systems, by considering the magnitude and source of six workload-related factors. [13, 14] The Spanish version of the NASA-TLX has been previously validated. [15, 16].

### Statistical analysis

The primary endpoint was powered to 80% based on (i) a two-sided z-test, (ii) type 1 error = 0.05, (iii) 30 procedures per group and that (iv) 30% of the procedures prior to the 4S program would require less than two sterilization cycles and 70% after the 4S program. The sample size chosen (30 procedures per group) was informed based on this hypothesis only. All study variables were analyzed descriptively. A two-sided z-test was used for the comparison for the primary endpoint. A p value < 0.05 was considered statistically significant. Fisher’s Exact test was used for categorical data and Wilcoxon rank sum test was used to compare the distributions of continuous data, unless otherwise specified. Analyses were conducted using R version 3.6.3. [19].

## Results

### Surgical set and procedure characteristics

In total, 60 procedures were observed: 30 with the current practice and 30 following the introduction of the 4S program. Table 3 shows a comparison of the type of sets used throughout the study. In line with introduction of the 4S program, only a Small Fragment set (DePuy Synthes, a Johnson and Johnson company) was used following the intervention, as opposed to the use of various procedure-specific sets used within the current practice. Table 3 also shows a comparison of anatomical area of surgery for the current practice, and following the introduction of the 4S program. With the current practice, nearly half of the procedures (46.7%) were ankle procedures whereas following the introduction of the 4S program, 26.7% of the procedures were ankle and 26.7% were clavicle (*p* = 0.020). There were no cancellations of the planned procedures in the study.

**Table 3:** Procedure characteristics for cohorts in the current practice and following introduction of the 4S program

	**Current practice (*n* = 30)**	**Following introduction of the 4S program (*n* = 30)**
**Set characteristics**		
Ankle	5 (16.7%)	0 (0.0%)
Elbow	4 (13.3%)	0 (0.0%)
PHILOS™	4 (13.3%)	0 (0.0%)
Small Fragments	15 (50.0%)	29 (96.7%)
Proximal tibia	2 (6.7%)	0 (0.0%)
Missing data	0 (0.0%)	1 (3.3%)
**Anatomical area**		
Ankle	14 (46.7%)	8 (26.7%)
Carpus	2 (6.7%)	0 (0.0%)
Clavicle	1 (3.3%)	8 (26.7%)
Elbow	2 (6.7%)	1 (3.3%)
Foot	0 (0.0%)	1 (3.3%)
Radius	1 (3.3%)	0 (0.0%)
Shoulder	7 (23.3%)	6 (20.0%)
Tibia	3 (10.0%)	2 (6.7%)
Other	0 (0.0%)	4 (13.3%)

### Primary outcome

With the current practice, only 30.0% of procedure sets were sterilized within one cycle, compared to 100.0% after the introduction of the 4S program (*p* < 0.001).

### Secondary outcomes

The introduction of the 4S program resulted in a reduction in the mean number of sterilization cycles required per procedure from 2.1 to 1.0 (*p* < 0.001). The maximum number of sterilization cycles per procedure for a single set with the current practice was 4, compared to 1 following the introduction of the 4S program.

The introduction of the 4S program resulted in a reduction in the mean set processing time of 24.1% in the OR (5.7 min, *p* = 0.040) and 35.3% (5.3 min, *p* = 0.005) in the sterilization department. No significant differences in the set processing time in the purchasing department were noted as a result of the intervention (Table 4). Overall, there was a significant difference in the total set processing time, with the overall mean turnover time for sets being reduced by 20.7% (10.5 min, *p* = 0.014) following the introduction of the 4S program.

**Table 4:** Set processing times for cohorts with the current practice and following the introduction of the 4S program

Set processing time in minutes, median (range)	A: Current practice (*n* = 30)	B: Following introduction of the 4S program (*n* = 30)	*p* value^a^
Overall turnover time	48.6 (16.3–94.1)	39.4 (9.6–70.8)	0.014
In operating room	22.1 (3.9–49.4)	17.5 (6.4–28.0)	0.040
In sterilization department	12.3 (1.9–42.0)	8.4 (3.2–30.0)	0.005
In purchasing department	12.6 (0.0–23.9)	11.8 (0.0–31.5)	0.842

^a^Kruskal-Wallis rank sum test

With the current practice, an implant was unavailable or damaged in 40% of procedures, compared to 3.3% following the introduction of the 4S program (*p* = 0.001).

The mean number of baskets placed into the cleaning machines per procedure per cycle was numerically lower following introduction of the 4S program compared to the current practice (5.17 versus 4.37, respectively, *p* = 0.121). The mean number of cleaning cycles per procedure was similar with the current practice and following the introduction of the 4S program (0.87 and 0.97, respectively; *p* = 0.165). The introduction of the 4S program significantly reduced the mean assembly time of containers for sterilization per procedure by 43.3% (4.22 min, *p* = 0.001). There was a significant difference in the mean number of containers entering the autoclave per procedure per cycle, which was reduced from 14.2 containers with the current practice, to 5.6 following the introduction of the 4S program (*p* < 0.001).

**Fig. 5:**
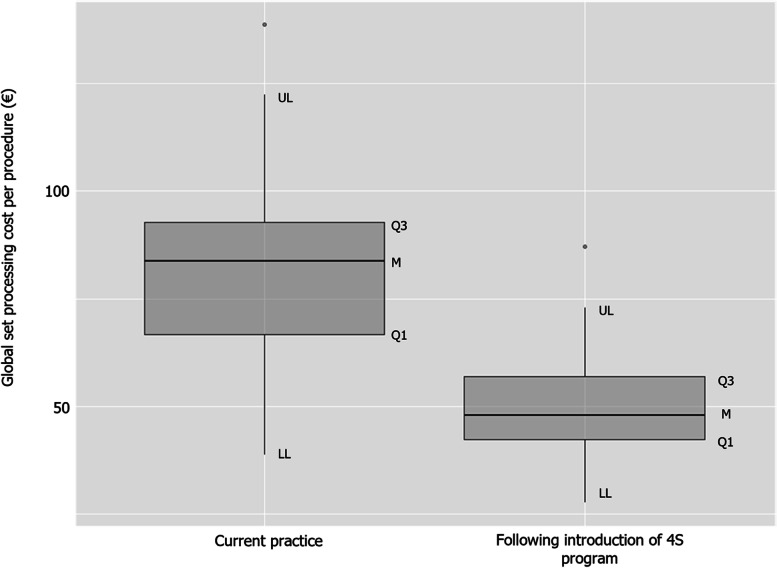
Global set processing costs in the current practice and following the introduction of the 4S program. Abbreviations: LL, lower limit; M, median; Q1, 25% quartile; Q3, 75% quartile; UL, upper limit. Q3 + 1.5×IQR (UL); Q1—1.5×IQR (LL). Points outside the box-and-whisker plot are outliers

The introduction of the 4S program was also associated with a reduction in processing costs, water consumption, and electricity consumption. The mean global set processing cost (OR, sterilization and purchase departments) within the current practice was €81.23 (USD$85.59), compared to €50.30 (USD$53.00) following the introduction of the 4S program (*p* < 0.001; Fig. 5; all currency conversions were conducted in June 2022 using the currency rate €100.00 = USD$105.37).

Mean values for the water and electricity consumption of the cleaning machines and steam autoclaves were obtained and used to calculate water and electricity usage within the current practice and following the introduction of the 4S program. Introduction of the 4S program was associated with reductions in mean water and electricity usage per procedure of 320 L (*p* < 0.001) and 5.77 kW (*p* < 0.001), respectively. These translated into cost savings per procedure of €0.61 (USD$0.64) and €0.70 (USD$0.74), respectively.

The NASA-TLX results from five staff members showed that mental, physical and time demands, along with performance and frustration levels, were all significantly improved with the 4S program (all *p* < 0.01; Table 5). There was also an improvement in the effort dimension following the program introduction, although this was not statistically significant (*p* = 0.753).

**Table 5:** Summary of results of NASA-TLX questionnaires for the current practice, and following introduction of the 4S program

NASA-TLX dimension, median score^a^ (range)	Current practice (*n* = 5)	Following 4S program introduction (*n* = 5)	*p* value^b^
Mental challenge	65 (50–80)	15 (15–40)	0.008
Physical demand	65 (45–80)	15 (15–40)	0.008
Time requirements	80 (65–90)	15 (15–40)	0.008
Effort^c^	65 (35–75)	40 (15–90)	0.753
Performance^d^	85 (55–85)	30 (15–40)	0.007
Frustration level	70 (55–90)	15 (10–40)	0.009

^a^Median score measured on a scale of 0–100 (very low–very high)

^b^Wilcoxon rank sum test

^c^Effort is defined as the degree of mental and physical effort that the individual has to make to obtain their level of performance

^d^Performance reflects the mental workload that an individual experienced to be satisfied with their performance

The seven satisfaction questionnaire respondents comprised three employees from the sterilization department, two from the OR and two from the purchasing department; these staff were present both before and after the introduction of the 4S program. The three sterilization staff reported that with current practice, it was necessary to re-sterilize sets that had not been fully used in surgical procedures 3–6 times per week, compared to 1–2 times following the introduction of the 4S program. All three staff members felt that it was “very important” to both reduce the weight of surgical sets and optimize the number of instruments per set. With the current practice, the difficulty of implant traceability was noted as “extremely difficult” to “difficult”. Ease of traceability improved following the introduction of the 4S program, with staff rating the difficulty level as “somewhat difficult” to “not difficult”.

Two staff members from the OR who completed the questionnaire both rated their current satisfaction levels with the overall process as “very satisfied” following the introduction of the 4S program, compared to “dissatisfied” when assessed within the current practice.

Staff in the purchasing department reported that human errors or misinterpretation in the OR report “often” led to subsequent administrative issues prior to the intervention, compared to “often” or “sometimes” after the introduction of the 4S program. Current satisfaction levels with the inventory management processes were reported to be “satisfied” and “neutral” following the program introduction and “dissatisfied” and “neutral” for the current practice.

## Discussion

Management of perioperative processes was improved based on the four pillars of the 4S program: 1) a standardized inventory, 2) a sterile implant portfolio, 3) a barcode system that enables a digital safety certification, and 4) a digital service for restocking. Its introduction in the perioperative process management of surgical sets for ORIF trauma surgeries resulted in a reduction in set processing times, number of sterilization cycles, and processing costs. Furthermore, following the introduction of the 4S program, staff satisfaction was increased and there was a positive sustainability effect due to the reduction in water and electricity usage.

A key improvement noted after the introduction of the 4S program was the increased speed of set processing. Following the 4S program introduction, total time spent in the OR, sterilization and purchasing departments decreased by 10.5 min, freeing up staff to undertake other tasks. Over 1,000 surgeries, this equates to a time saving of 174 h. The removal of all implants (screws and plates) from the sets leads to a reduction in size and variability of sets, thereby optimizing inventory management and allowing for the use of one central depository per OR. Surgeons may have concerns about the additional time burden of opening separately packaged implants, thus increasing OR time. However, a hospital in Germany assessed how the 4S program impacted surgical set management practices in trauma surgeries. It was reported that there was no significant difference in median incision-to-suture time after the change in surgical set management practice, and a numerical reduction of 4 min following introduction of the 4S program, indicating that separately packaged implants and screws did not confer an additional time burden during surgery, contrary to the authors' initial expectations. [20].

The introduction of a stock management system and the digitalization of supply chain procedures such as consumption, purchasing and invoicing, aims to reduce the timings in the purchasing department. However, in this study, timings reported for the purchasing department were not significantly decreased following introduction of the 4S program. One possible explanation for this could be the reduced productivity of one employee who suffered an injury (not related to the study) in the period after the 4S program was introduced. Another explanation might be that the 4S stock management tool (eSims) was not fully integrated with the hospital’s enterprise resource planning (ERP) system. System integration may have eliminated all manual transactions in the hospital’s ERP system, since each report would have been automatically sent to the system instead of being sent via email to the responsible team.

The provision of pre-sterilized implants separately to the surgical instrument sets reduces the number of sterilization cycles required in the hospital and results in fewer containers requiring sterilization per procedure, reducing the workload for sterilization staff. Sterilized surgical sets no longer need to be opened to add new or missing implants, thereby avoiding unnecessary reprocessing and further reducing the burden on hospital staff.

The combination of decreased set processing times and the requirement for fewer sterilization cycles translates into measurable cost savings. Analysis found that the whole process was €30.93 (USD$32.59) more expensive within the current practice compared to the process following the introduction of the 4S program. Over 1,000 procedures, the mean process cost saving was calculated to be €30,930 (USD$32,590). This may be of particular importance for healthcare systems, given the growing concern over the increasing rates of healthcare expenditure in Spain and other developed countries. [21].

Reduced environmental impact is another important outcome of the introduction of the 4S program. The United States' healthcare system and has the world’s highest healthcare emissions, both in absolute and per capita terms, producing 1.72 metric tons of CO_2_ per capita. [22] Spain’s healthcare footprint, in comparison, is estimated to be 0.36 metric tons of CO_2_ per capita, a smaller but still substantial contributor to overall emissions. [22].

Hospitals have a key role to play in addressing resource consumption levels and must find ways to reduce their carbon footprint. [24] Studies have shown that ORs are the most resource intensive area in the hospital, with surgical instruments being the main driver of the environmental impact of surgical procedures. [25–27] Several studies looking into ways to reduce waste and carbon footprint in hospitals and in the OR have found that simple changes can have a significant impact. [6, 28] For example, simply reducing the number of trays used in the OR, and thus the amount of tray wrapping used, leads to a reduction in waste. [6] In this study, the introduction of the 4S program reduced the number of sterilization cycles required by more than 50% and reduced the number of containers entering the autoclave per procedure, leading to a reduction in water and electricity consumption, resulting in a positive environmental impact in the sterilization department. Over 1,000 procedures, the water usage is calculated to be reduced by 320,000 L and electricity usage by 5,770 kW, resulting in potential cost savings of €608 (USD$640) and €701 (USD$739), respectively.

The final improvement observed was in staff satisfaction. The new standardized sets in the 4S program are much lighter than those typically used (6 kg versus approximately 12 kg), making them easier for OR personnel to transport and set up, and potentially reducing the risk of injury from transporting heavy sets. With the standardization of sets, there are fewer procedure-specific sets which simplifies the overall process. Carrying out inventory of every implant used is also easier, since surgical personnel only need to read the barcode label on the packaging. These improvements were captured in the results of the NASA-TLX and the questionnaire. Staff reported reduced physical and mental demands and an overall reduction in workload following introduction of the 4S program. They also noted that it was easier to trace implants following introduction of the program and that fewer administrative errors occurred in the purchasing department. The utilization of barcoding allows for tracking of implants, increases the ease with which hospitals can comply with MDR traceability requirements and simplifies the purchasing process as single items can be easily identified and reordered. Digitalization of the stock management results in the immediate flow of information from the OR to the administrative teams, with enhanced security compared to paper-based methods which are more easily damaged or lost.

This study was performed in Spain, where there has been a recent increase in the proportion of hospitals using sterile instruments and implants. In the authors’ experience, in 2019 only six departments in Spain were operating with sterile osteosynthesis material; by 2022 there were 44. It is therefore likely that wider implementation of the 4S program would be beneficial.

The findings of our study are in line with published literature in that several studies have reported measurable time and cost savings when changes are made to the management of surgical sets. Introduction of a perioperative management program for elective orthopedic surgeries, which involved the optimization of surgical tray contents, resulted in a reduction in instrument processing time and associated costs. [9] A review of surgical sets used in otolaryngology surgeries found that the removal of unused instruments could reduce set size by approximately 60%, which would improve operating room throughput and impact cost containment. [6] Moderate cost-savings have been reported in a cost-analysis study of the streamlining of instrument trays for otolaryngology procedures. [5] Resource savings have been shown to not be limited to adult surgeries. A systematic review of the standardization of surgical sets in pediatric surgical cases found costs were reduced, without an observable impact on OR time or safety. [10].

This study has some limitations. A convenience sample was used, and although this sampling method is commonly used in clinical trials and observational studies, the sample may not be representative of the total population. Any inferences made in this study are limited to the data presented and may not be generalizable to other settings. As the intervention received (before or after the introduction of the 4S program) was based on a convenience sample of consecutive cases and not randomly assigned, it is only possible to infer correlation between the intervention and outcomes, again limiting the conclusions that can be drawn from this study. Additional work will be required to test the hypothesis that the 4S program drives efficiency and cost savings in other settings.

Only procedure-related data were collected during the study, and no patient baseline characteristics were recorded, e.g. comorbidities. It was therefore not possible to control for potential confounders in the analyses. Additional confounding factors that may have influenced surgical set processing were not recorded but had the potential to have a significant impact on findings (e.g. the additional time required if one instrument was missing/damaged). In-person observation of OR staff may have influenced behavior and subsequent outcomes (e.g., via the Hawthorne effect). [29] Surgeons were not blinded to the intervention given the nature of the study design which may have introduced measurement bias, though this may be mitigated as primary and secondary endpoints were objectively assessed by the sterilization department. We did not encounter events independent of the surgeons' practice that may have led to additional sterilization cycles following the introduction of the 4S program, which may be the result of the limited sample size.

Although the surgical procedures remained consistent throughout the study, patient outcomes were not recorded. In future studies, it would be valuable to collect data on surgical and safety endpoints for patients. Whilst the program introduction described here is specific to DePuy Synthes Trauma (a subsidiary of Johnson & Johnson), it is our belief that the principles underpinning the 4S program are nonetheless transferable to other surgical specialties to realize similar benefits from the perspective of reduced resource use and costs, and improved sustainability of these procedures. Further research would be advisable to understand the potential benefits after a complete integration of the stock management tool.

Key strengths of this study include the prospective study design and broad patient inclusion criteria, mitigating selection bias, and standardized data collection instruments. The same hospital staff were present for procedures using the current practice and for those following the introduction of the 4S program, thereby reducing variability in potential confounding factors, despite the necessary pause of the study during the COVID-19 pandemic.

To our knowledge, this is the first study evaluating time management, cost savings and the environmental impact of streamlining perioperative management of surgical sets via the 4S program in trauma surgery. Further research should be undertaken, to demonstrate whether the positive impact of the broad and comprehensive changes made to surgical set management demonstrated in this single-center study in Spain, focusing on ORIF surgeries, is generalizable to other surgical specialties across the world.

## Conclusions

In conclusion, this study demonstrated substantial time and cost savings that could be achieved by the introduction of the 4S program. Furthermore, the 4S program may contribute to net positive environmental impact and results in increased staff satisfaction. The introduction of a lean management program such as the 4S approach is of paramount importance, given the shift towards a value-based approach to management within healthcare systems and the necessity to minimize costs. Scaling the 4S program to other specialties and settings may improve the management of surgical instruments and implants, resulting in lower costs for hospitals, payers, and ultimately, patients.

## Data Availability

The data that support the findings of this study are available from the corresponding author upon reasonable request. More details on Johnson & Johnson's commitment to transparency are available via the following link: https://www.jnj.com/coronavirus/our-commitment-to-transparency/.

## Abbreviations

AEAS-AGA: Asociación Española de Abastecimientos de Agua y Saneamiento—Asociación Española de Empresas Gestoras de los Servicios de Agua Urbana
ERP: Enterprise resource planning
EU: European Union
kg: kilograms
kW(h): kilowatt (hour)
L: liter
MDR: Medical Device Regulations
NASA-TLX: NASA Task Load Index
OR: Operating room
ORIF: Open reduction internal fixation
SQUIRE: Standards for Quality Improvement Reporting Excellence
USD: United States Dollar
